# Evaluation of Ocular Perfusion in Patients with End-Stage Renal Disease Receiving Hemodialysis Using Optical Coherence Tomography Angiography

**DOI:** 10.3390/jcm12113836

**Published:** 2023-06-03

**Authors:** Larissa Lahme, Jens Julian Storp, Elena Marchiori, Eliane Esser, Nicole Eter, Natasa Mihailovic, Maged Alnawaiseh

**Affiliations:** 1 Department of Ophthalmology, University of Muenster Medical Center, 48149 Muenster, Germany; larissa.lahme@ukmuenster.de (L.L.);; 2 Department of Vascular and Endovascular Surgery, University of Muenster Medical Center, 48149 Muenster, Germany; 3 Department of Ophthalmology, Klinikum Bielefeld gem. GmbH, 33604 Bielefeld, Germany

**Keywords:** renal disease, optic nerve, macular perfusion, flow density, vessel density, foveal avascular zone

## Abstract

Hemodialysis (HD) is known to affect ocular blood flow. This case-control study aims to evaluate macular and peripapillary vasculature in patients with end-stage renal disease (ESRD) receiving HD in comparison to matched controls. A total of 24 eyes of 24 ESRD patients receiving HD and 24 eyes of 24 healthy, age- and gender-matched control subjects were prospectively included in this study. Optical coherence tomography angiography was used to image the superficial (SCP), deep (DCP), and choriocapillary (CC) macular vascular plexus, as well as the radial peripapillary capillaries (RPC) of the optic disc. In addition, retinal thickness (RT) and retinal volume (RV) were compared between both groups. Flow density (FD) values of each retinal layer and data of parameters related to the foveal avascular zone (FAZ), as well as RT and RV, were analyzed using Mann–Whitney U tests. There was no significant difference in FAZ parameters between the two groups. Whole en face FD of the SCP and CC was noticeably reduced in the HD group in comparison to the control group. FD was negatively correlated with the duration of HD treatment. RT and RV were significantly smaller in the study group than in controls. Retinal microcirculation appears altered in patients with ESRD undergoing HD. Concurrently, the DCP appears more resilient towards hemodynamic changes in comparison to the other microvascular retinal layers. OCTA is a useful, non-invasive tool to investigate retinal microcirculation in ESRD patients.

## 1. Introduction

Altered ocular perfusion has been described in various systemic diseases [1,2]. In particular, end-stage renal disease (ESRD) has been reported to be associated with distinct changes in cardiovascular and ocular perfusion [3,4,5,6,7,8]. The objective of hemodialysis (HD) is to stabilize the kidneys’ excretory function. The ultrafiltration used in this process influences the hemodynamic status of the patient to such an extent that ocular circulation can be affected [9,10].

The retina offers the unique ability to conduct an in vivo assessment of the microvascular tissue, the significance of which has previously been highlighted for its transferability to the renal status [3,4,8]. At the same time, vascular imaging options for ESRD patients are strongly limited. Multiple factors, such as the necessity for contrast medium administration, cost-effectiveness, and the possibility to quantify and compare imaging results over time pose a challenge towards adequately monitoring patients. The complexity and high intensity of therapy combined with impaired renal function additionally limit imaging options. Optical coherence tomography angiography (OCTA) represents a novel development in vascular imaging of the retina and optic disc. It allows for the non-invasive visualization of blood vessels through the detection of moving blood cells and is being discussed to act as a possible surrogate biomarker to estimate microvascular status [11]. To date, only a limited number of studies have investigated capillary changes in patients receiving HD using OCTA [12,13,14]. These trials have assessed short-term changes in OCTA parameters either before and after HD sessions, or over the course of individual HD sessions, focusing on relative alterations between same-day measurements, rather than on the overall effect of HD on ESRD patients. Recent trials have demonstrated a reduction in flow density (FD) in patients with renal impairment in comparison to healthy controls [15,16,17]. Being the main treatment option for patients with ESRD, HD is expected to have an impact on retinal vasculature in OCTA imaging. Currently, the impact of HD on the individual layer of the retinal microvascular plexus and the long-term effect of HD treatment on retinal OCTA measurements remain unclear.

The primary aim of this study is to evaluate differences in retinal FD in patients with ESRD receiving HD treatment in comparison to a matched control population, the hypothesis being that FD might be altered between the study and control groups. As a secondary aim, the relationship between the duration of HD therapy and alterations in OCTA parameters will be investigated, in order to evaluate long-term effects of HD treatment on OCTA metrics. Thirdly, differences in retinal thickness and volume between both groups are investigated and monitoring capabilities of OCTA for ESRD patients are discussed.

## 2. Materials and Methods

### 2.1. Design and Setting

This prospective, monocentric case-control study adhered to the “Strengthening the Reporting of Observational Studies in Epidemiology (STROBE) Statement” guidelines and was conducted at the Departments of Ophthalmology and Vascular and Endovascular Surgery at the University of Muenster Medical Center, Germany. Patients with ESRD, who were treated with HD, were consecutively enrolled in this study. Similarly, a group of healthy controls were drawn into the study for comparative analyses. The study followed the tenets of the Declaration of Helsinki and was approved by the Ethics Committee of the University of Muenster, North Rhine Westphalia, Germany.

### 2.2. Patient Examination

All participants received a thorough ocular examination which included IOP (intraocular pressure) evaluation, refraction measurements, slit lamp biomicroscopy, funduscopy, and OCTA imaging. Before participation in the study, the study protocol was explained in detail to all participants and they signed an informed consent form. For each patient in the study group, one age- and gender-matched person was included in the control group. All subjects included in this study had to be over 18 years of age. Patients in the study group had to meet the following inclusion criteria: they had to have terminal renal insufficiency, to be receiving HD therapy three times weekly, and were further required to have started HD treatment at least 6 months prior to study enrollment. Individuals of the control cohort were required to not have any retinal, papillary, or neurological pathologies. In addition, they were required to not have or have had any renal disease or other systemic illness besides arterial hypertension or diabetes mellitus without ocular manifestation.

Individuals with media opacities that prevented high-quality imaging, such as significant cataract, or who had vitreoretinal or corneal disease, had history of vitreoretinal or corneal surgery status, or who suffered from any neurological disease, were not eligible for study inclusion. Furthermore, individuals with any signs of ocular diseases were excluded from the study. The principle of OCTA has been described in detail elsewhere [11]. Briefly, repeated OCT scans of a certain area are performed and the resulting images of that area are evaluated to identify possible changes occurring between acquisitions. Blood flow in retinal vessels causes differences between successive OCT images, whereas static tissue does not [11]. We performed OCTA of both the macula and the peripapillary area, as previous studies have shown that systemic factors affecting retinal perfusion may influence both areas [18,19,20]. The spectral-domain (SD) RTVue XR Avanti system was used for OCTA imaging (Angiovue/RTVue-XR Avanti, Optovue Inc., Fremont, CA, USA). Eyes were imaged without topical dilatation. 3 × 3 mm^2^ scans were used for angiographic imaging of the macula, 4.5 × 4.5 mm^2^ scans were conducted for papillary scans. The AngioVue algorithm automatically calculated FD, which equals the ratio of bright pixels to the total number of pixels per scan and is reported as a percentage value (%), for different retinal layers and sublocations.

OCTA imaging was performed by an expert examiner under the same conditions in the same location. All patients received the OCTA measurement on a day they were not receiving dialysis between two days of dialysis therapy. Patients were required to rest for at least five minutes prior to OCTA imaging in order to rule out the effects of blood pressure and heart rate changes on OCTA imaging [21]. Each macular and papillary slab was imaged at least three times in a row. Among the three generated images, the one with the highest quality index (QI) was chosen to go to the next step of image selection. If two or more images had the same QI, one was chosen at random by coin flip. Next, using coin flip, it was decided which of the patient’s eyes (left, right) was to eventually be included in statistical analysis. In the end, only one eye per patient was drawn into the study. Scans containing artifacts or missing data were rejected. Images were required to have a QI of ≥7 and a signal strength index (SSI) of ≥50.0. If necessary, manual sector segmentation was carried out. Parameters of the foveal avascular zone (FAZ) were calculated automatically by the AngioVue software (version 2016.1.0.26) on the basis of macular scans.

### 2.3. Comparison of Measurements

**Figure 1:**
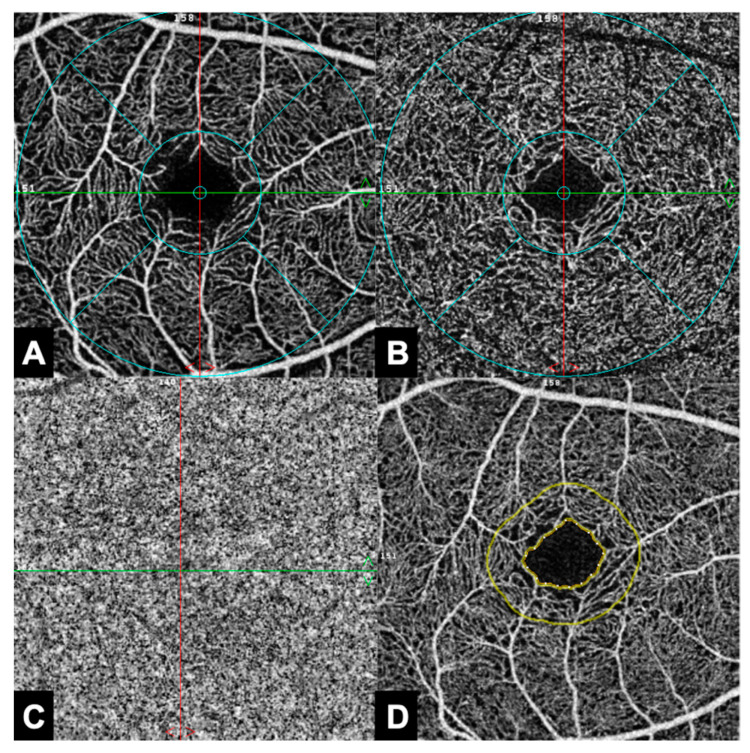
Exemplary imaging of the analyzed macular regions in optical coherence tomography angiography (OCTA) in en face view. (**A**): Superficial capillary plexus (SCP). (**B**): Deep capillary plexus (DCP). (**C**): Choriocapillaris (CC). (**D**): Foveal avascular zone (FAZ) and the area surrounding it in a distance of 300 µm (FD-300).

**Figure 2:**
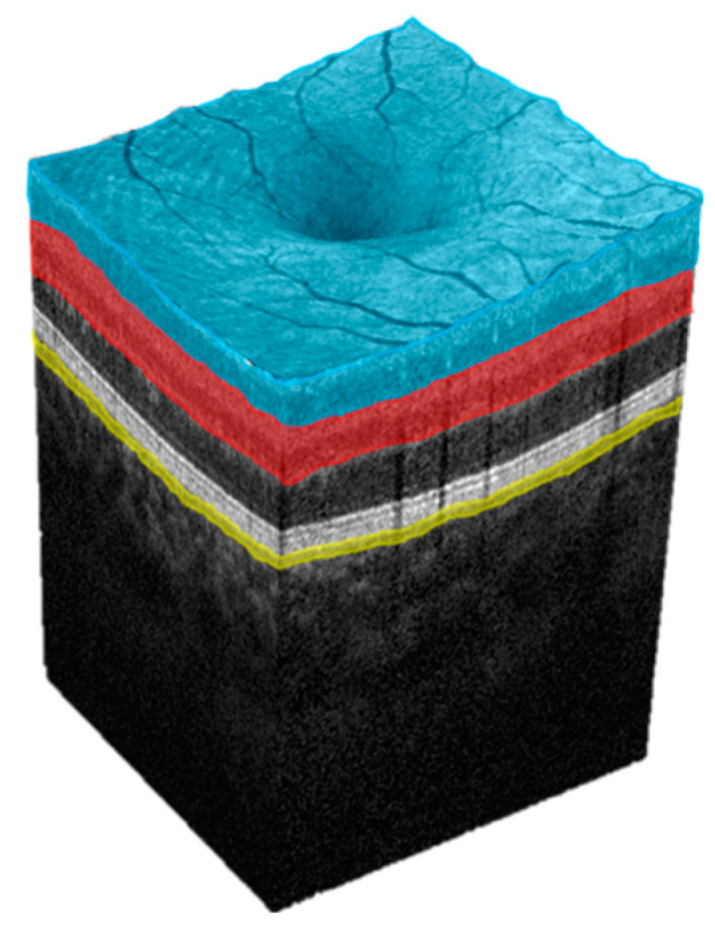
Approximated tomographic representation of the macular layers analyzed in this study. Note that the layer-specific subsectors analyzed in this study are not displayed here. Blue: superficial capillary plexus (SCP). Red: deep capillary plexus (DCP). Yellow: choriocapillaris (CC).

**Figure 3:**
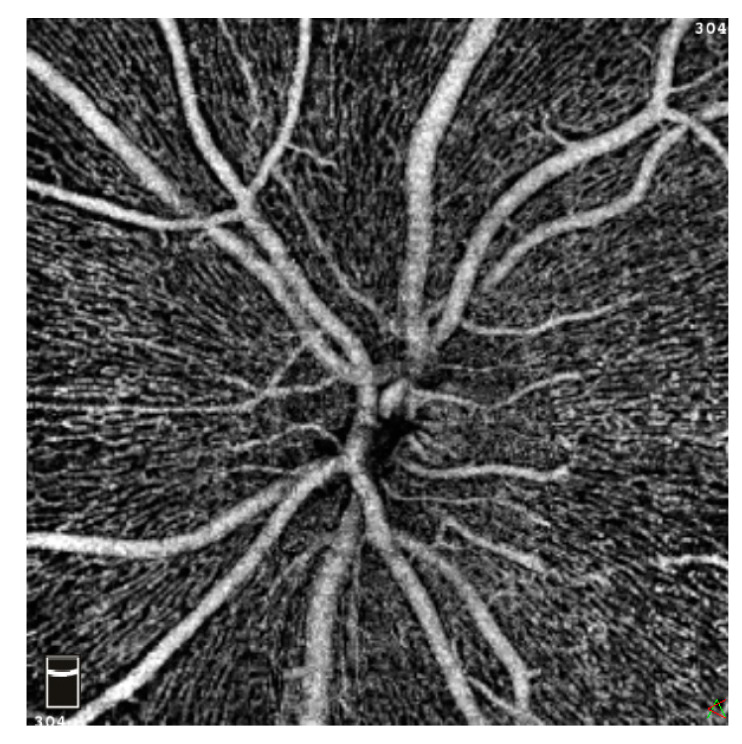
Exemplary imaging of the papillary region in optical coherence tomography angiography (OCTA) in en face view.

Measurement results of patients receiving HD were compared to measurement results of a healthy age- and gender-matched control cohort. In total, 17 parameters related to the superficial (SCP), deep (DCP), and choriocapillary (CC) plexus of the macula, the radial peripapillary capillaries (RPC), and FAZ were extracted and analyzed. The FAZ parameters, including FAZ area, FAZ perimeter, acircularity index (ACI), and FD-300, were automatically segmented by the AngioVue software. ACI represents the ratio as to which degree FAZ equals the symmetry of a perfect circle, with the value 1.0 representing a perfect circle. FD-300 displays the capillary density in a 300 µm radius around the FAZ [22]. Figure 1 and Figure 2 illustrate the individual retinal layers from an en face perspective (Figure 1) and from a three-dimensional tomographic perspective (Figure 2). Figure 3 shows OCTA imaging of the papillary region (Figure 3). FD values were retrieved from the en face images and further correlated with HD duration as a secondary objective.

In addition, as a tertiary objective, the total retinal thickness (RT), equaling the average retinal thickness over the entire OCT scan, as well as the total retinal volume (RV), equaling the average retinal volume over the entire OCT scan, were extracted from the images generated by the AngioVue system. Total RT and RV were compared between the study group and control group.

### 2.4. Statistical Analysis

A post-hoc analysis was performed using G*Power 3.1 (Heinrich-Heine-Universität Düsseldorf, Germany, www.gpower.hhu.de (accessed on 11 May 2023). For 24 patients in each group, and an α of 0.05, we determined a power of 0.67. Data were recorded in the spreadsheet software Microsoft Office Excel 2010, version number 14.0 (Microsoft, Redmond, WA, USA). Statistical analysis was performed using SPSS, version 28.0 (IBM SPSS Statistics). Data distribution was tested using the Shapiro–Wilk test. The data did not fit a normal distribution. Therefore, OCTA measurements were analyzed using Mann–Whitney-U tests and Spearman’s correlation coefficient was applied to investigate the degree of correlation between the duration of HD treatment at the time of OCTA imaging and the OCTA data (rSp). Due to the exploratory nature of the study, no adjustment was made for multiple testing. All analyses are explorative and should be interpreted accordingly. All *p* values ≤ 0.05 were considered statistically noticeable.

## 3. Results

In total, 24 eyes of 24 patients receiving HD and 24 eyes of 24 healthy age- and gender-matched individuals were included in the study. All individuals of the HD group suffered from ESRD. ESRD patients had received HD treatment for a median duration of 3.8 years at time of study inclusion. Characteristics of the study population are summarized in Table 1.

Image quality-related parameters did not differ significantly between the two cohorts. Likewise, there were no differences between both cohorts in regards to visual acuity or spherical equivalent (Table 1).

All patients with diabetes mellitus received regular blood sugar treatment and had no signs of diabetic retinopathy in any of either eye at time of study enrollment. All individuals of the study group received HD treatment due to ESRD. The underlying causes that led to CKD were: hypertensive nephropathy (n = 11), polycystic kidney disease (n = 4), cirrhotic kidney disease (n = 2), chronic glomerulonephritis (n = 2), interstitial nephritis (n = 2), focal segmental glomerulosclerosis (n = 1), nephroangiosclerosis (n = 1), and nephroblastosis (n = 1).

### 3.1. Primary Objective

Apart from the superficial foveal sector, the other sectors of the SCP showed noticeably reduced FD values for the study group in comparison to the control group. At the level of the DCP, no noticeable difference between the two groups was found. In contrast, FD of the CC was noticeably reduced in all investigated sectors. Though the whole en face and peripapillary sectors of the RPC showed higher absolute FD values in the control group, the sectors of the RPC were statistically indifferent between both cohorts. Meanwhile, there were no significant differences in any of the FAZ-related parameters between the two groups, except for FD-300 length density (Table 2).

### 3.2. Secondary Objective

The majority of the SCP and RPC, as well as all sectors of the CC, were noticeably negatively correlated with the duration of HD therapy at the time of OCTA imaging, illustrating that a longer period of HD treatment was associated with a decrease in FD in these regions. This was also seen for the foveal region of the DCP and the capillaries adjacent to the FAZ (FD-300) (Table 3).

### 3.3. Tertiary Objective

Total RT and RV were significantly smaller in patients receiving HD than in controls (Table 4).

## 4. Discussion

The influence of HD on the systemic and cerebral vascular system is manifold. Previous studies have demonstrated altered cerebral perfusion in patients receiving dialysis [23]. Patients undergoing HD or peritoneal dialysis tend to have a 2.5 times or higher probability of having moderate to severe cognitive impairment [24,25] and are at increased risk to suffer from cerebral stroke during the first year on dialysis [26,27,28,29]. Isshiki et al. also demonstrated that the reduction of the cerebral blood flow in patients with HD is correlated with the duration of the dialysis and that a reduction of cerebral blood flow was found irrespective of clinical symptoms or mini mental state examination score [24]. Patients with ESRD receiving renal replacement therapy are at a four-fold to ten-fold higher risk for experiencing stroke relative to the general population, and stroke risk increases by a factor of seven-fold during the initial year of dialysis [26,27,28,29]. While the exact etiology of these findings remains unclear, most authors attribute these observations to changes in microvascular perfusion due to changes in hemodynamics.

It has been shown that ESRD itself also accelerates vascular aging and leads to increased vascular calcification [30,31]. Yong et al. compared FD in OCTA between patients with reduced renal function and healthy controls. FD was observed to be reduced in the group of patients with renal impairment [16]. Various authors describe a negative correlation between OCTA-derived FD values and renal function [15,32,33]. However, it remains unclear whether this correlation is still visible in patients receiving HD treatment.

Monitoring of the microperfusion is often difficult to realize in clinical practice. OCTA enables a noninvasive evaluation of these changes in an area covering the retina, optic disc, and (in parts) the choroid. It provides high-resolution images of the retinal vasculature and allows for the quantitative analysis of the retinal microcirculation. The assessment of quantitative OCTA metrics shows good repeatability in healthy subjects and in patients with different ocular diseases [34,35].

To our knowledge, and after intensive literature search, this study is the first to show that FD is significantly negatively correlated with the total duration of dialysis therapy. Former studies investigating retinal microvasculature using OCTA have tracked relative changes in FD and FAZ features over the course of one HD session, but have not analyzed the long-term effect of permanent dialysis therapy on OCTA metrics. This study represents the first to investigate this issue. Most OCTA studies currently available in the literature are restricted to analyzing capillary and structural changes in patients directly before and after an HD treatment session, comparing OCTA measurements taken only hours apart. The findings reported in these studies remain inconclusive. Zhang et al. conducted SD-OCTA measurements in 77 patients suffering from ESRD before and after HD treatment [13]. The authors describe a decrease in FD in the outer retina. However, there was no change observed in the SCP, DCP, or CC. They attribute the outer retinal changes to a reduced vasculature and deficient autoregulatory control in these patients. This is in line with the findings by Shin et al., who did not see any differences in FD of the SCP and DCP before and after HD sessions, but who describe the FD of the CC to decrease significantly after the treatment [12]. Further trials have generated different findings, as they report a reduction of FD at the level of the DCP and an increase in FD in the SCP for hypotensive patients [14]. Fursova et al. described a decrease in the circularity index of the FAZ associated with HD treatment [32]. We found no significant difference in regards to FAZ related parameters between ESRD patients treated with HD and control patients. This might be attributable to different amounts of time spent on HD treatment between various studies. It cannot be ruled out that with increasing time spent on treatment, FAZ parameters of ESRD patients receiving HD therapy might start to differ more noticeably in comparison to controls.

In this study, ESRD patients with HD treatment had a reduced FD in the SCP and CC and a trend towards reduced FD values in the peripapillary RPC scans in comparison to healthy subjects. The differences in the DCP between both cohorts were insignificant. It is known that OCTA imaging in the DCP is more challenging and affected by projection artefacts and therefore the repeatability of FD data in the DCP was found to be weaker compared with that of the superficial retinal OCT angiogram [36,37,38]. We assume that in contrast to SCP and CC, the DCP not showing significant differences in FD values in this study can be attributed to a more potent autoregulatory control in this retinal layer. As Zong et al. have demonstrated, the autoregulatory response of the retina can differ between anatomical locations [39]. Histopathological studies in the rat have demonstrated that autoregulatory responses of the retina differ between its vascular layers [40]. In this regard, the observation made in our study suggests that the DCP, in comparison to the other capillary plexus, is more resilient towards hemodynamic changes. Similarly, other studies have shown that patients with Alzheimer’s disease or carotid stenosis have reduced flow density in the SCP but not in the DCP of the macula [18,19].

Further reports on the effect of HD on the RT in OCT trials are also inconclusive. While some authors report no effect of dialysis on total RT [12,41,42,43], others report a thinning of the retina between the measurements before and after a HD treatment [13,44]. It is important to note that comparability to these studies is limited, as they report changes observed immediately before and after HD treatment, whereas in this study patients were imaged on a day they were not receiving dialysis. Nevertheless, the findings in this study are comparable to the results of Theodossiadis et al., who report a reduction of total RT and RV in eyes with and without macular edema after HD. Applying swept-source OCTA, Shin et al. were even able to comment on changes related to the choroid. They report that choroidal thickness decreased significantly after treatment and correlated with the ultrafiltration volume [12].

Other studies have demonstrated that the extent of the effects seen in various organs and tissues correlates with the duration of HD therapy. Chrapko et al. have shown that the time spent on HD appears to significantly affect the cardiac sympathetic nervous system [45]. Isshiki et al. also showed a correlation between the duration of dialysis and the decrease in cerebral blood flow in HD patients [24]. These observations are in line with the findings of this study, which for the first time demonstrates a negative correlation for sectors of SCP, CC, RPC, and in parts the DCP (fovea) and FAZ (FD-300) with the duration of HD treatment. It remains unclear whether HD itself, or rather the underlying renal disease, has caused the reduction in retinal FD. It is probable that ESRD is the main cause of FD decrease, as it has been shown to be associated with reduction in ocular perfusion [15,32,33]. HD, being the main treatment method for ESRD patients, might have a positive influence on FD values and could therefore attenuate the effect that ESRD has on FD measurement results. Either way, HD should be considered a possible confounding factor in future OCTA trials.

### Limitations

Multiple parameters, such as cardiovascular risk factors known to cause alterations in retinal perfusion, have been reported in the literature. In this study, there was no noticeable difference in the number of patients with arterial hypertension, or diabetes mellitus. Nevertheless, these individual patient characteristics should be taken into account when interpreting the results presented in this and other OCTA studies. Future investigations should take these factors into account to reduce bias [46,47].

As the measuring of CC flow data is more challenging than the imaging of the inner retinal plexus, caution in the interpretation of CC data is advised. Future studies investigating the CC should carefully check images to ensure statistical reliability.

Furthermore, this study did not distinguish between different causes of ESRD. Further studies with a larger patient collective, a prospective design, a third group including patients with ESRD but without HD therapy, and a corresponding subgroup analysis are needed to replicate the findings reported in this case-control study.

## 5. Conclusions

In conclusion, patients with ESRD receiving HD show reduced total RT and RV, decreased FD in the SCP, CC and a trend towards a reduction of FD in the RPC in OCTA imaging. FD was furthermore negatively correlated with HD treatment in these regions. In contrast, the DCP appeared more resilient towards hemodynamic changes caused by HD treatment. HD should be regarded as a possible confounding factor when interpreting FD values and structural OCT results.

## Figures and Tables

**Table 1:** Characteristics of study population. Values are reported as median (25% quartile; 75% quartile). *p* values are displayed where applicable.

	Study Group	Control Group	*p* Value
Patients (n)	24	24	
Eyes (n)	24	24	
Age (years)	56.11 (49.77; 64.40)	56.86 (48.92; 65.71)	0.97
Gender (m: f)	13: 11	13: 11	
Laterality (r: l)	7: 17	9: 15	
Patients with arterial hypertension (treated; n; %)	21 (84%)	16 (67%)	
Patients with arterial hypertension (untreated; n; %)	0 (0%)	0 (0%)	
Patients who were active smokers at Time of study enrolment (n, %)	4 (16%)	3 (12%)	
Patients who were former smokers at Time of study enrolment (n, %)	2 (8%)	1 (4%)	
Patients with diabetes (n, %)	5 (20%)	5 (20%)	
Duration of HD treatment at time of OCTA imaging (months)	46.0 (31.0; 84.0)		
QI (macula)	8.00 (7.00; 8.00)	8.00 (7.00; 8.25)	0.33
SSI (macula)	64.53 (59.41; 67.89)	65.95 (62.34; 69.22)	0.11
QI (optic disc)	8.00 (7.00; 8.00)	8.00 (7.00; 8.00)	0.52
SSI (optic disc)	64.53 (59.41; 67.89)	65.39 (59.74; 68.94)	0.10
Visual acuity (LogMAR)	0.1 (0.0; 0.1)	0.1 (0.0; 0.1)	0.77
Spherical equivalent	0 (−1.59; 0.09)	0 (−0.69; 1.28)	0.32

n = number, m = male, f = female, r = right, l = left, HD = hemodialysis, QI = quality index, SSI = signal strength index, LogMAR = logarithm of the minimum angle of resolution.

**Table 2:** Results of statistical comparison of flow density (FD) values of hemodialysis patients (study group) to healthy control patients. Values are presented as median (25% quartile; 75% quartile). *p* values ≤ 0.05 are highlighted in bold.

Location	Parameter	Study Group	Control Group	*p* Values
SCP (%)	Whole en face	41.41 (38.92; 44.39)	45.00 (43.45; 47.15)	**<0.01**
	Fovea	16.29 (14.11; 19.61)	17.24 (14.86; 22.00)	0.29
	Para fovea	43.98 (40.82; 46.32)	47.74 (46.36; 49.91)	**<0.01**
DCP (%)	Whole en face	48.19 (45.49; 51.69)	49.23 (47.21; 51.34)	0.33
	Fovea	31.43 (26.74; 36.67)	33.71 (29.23; 38.41)	0.13
	Para fovea	49.77 (48.26; 53.03)	51.02 (48.63; 53.39)	0.42
CC (%)	Whole en face	65.96 (64.55; 68.82)	69.12 (65.81; 71.97)	**0.05**
	Fovea	65.51 (58.02; 69.22)	68.58 (66.38; 71.64)	**0.01**
	Para fovea	65.38 (63.65; 68.35)	68.47 (65.08; 71.74)	**0.05**
FAZ	FAZ area (mm^2^)	0.29 (0.20; 0.35)	0.28 (0.19; 0.32)	0.42
	Perimeter (mm)	2.11 (1.87; 2.40)	2.09 (1.68; 2.34)	0.38
	Acircularity index	1.17 (1.11; 1.18)	1.13 (1.10; 1.21)	0.41
	FD-300 area density	48.13 (43.90; 48.89)	48.68 (46.96; 49.82)	0.08
	FD-300 length density	16.38 (14.69; 17.42)	17.32 (16.54; 17.99)	**<0.01**
RPC (%)	Whole en face	54.20 (50.90; 56.14)	55.45 (53.80; 57.15)	0.09
	Inside disc	57.99 (55.74; 62.94)	59.88 (55.88; 61.95)	0.90
	Peripapillary	55.76 (51.59; 58.76)	57.45 (56.02; 60.10)	0.07

SCP = superficial macular capillary plexus, DCP = deep macular capillary plexus, CC = choriocapillaris, FAZ = foveal avascular zone, mm = millimeters, para fovea = area surrounding the fovea.

**Table 3:** Spearman correlations between the duration of HD treatment at time of OCTA imaging and FD. *p* values ≤0.05 are highlighted in bold.

Location	Parameter	Duration of HD Treatment at Time of OCTA Imaging	
		r Spearman	*p*-Value
SCP	Whole en face	−0.47	**<0.01**
	Fovea	−0.26	0.08
	Para fovea	−0.52	**<0.01**
DCP	Whole en face	−0.21	0.15
	Fovea	−0.31	**0.03**
	Para fovea	−0.16	0.28
CC	Whole en face	−0.39	**0.01**
	Fovea	−0.30	**0.04**
	Para fovea	−0.40	**0.01**
FAZ	FAZ area (mm^2^)	0.22	0.14
	Perimeter (mm)	0.25	0.09
	Acircularity index	0.21	0.16
	FD-300 area density	−0.29	0.05
	FD-300 length Density	−0.41	**<0.01**
RPC	Whole en face all	−0.37	**0.01**
	Inside disc all	−0.09	0.55
	Peripapillary all	−0.37	**0.01**

SCP = superficial macular capillary plexus, DCP = deep macular capillary plexus, CC = choriocapillaris, FAZ = foveal avascular zone, mm = millimeters, para fovea = area surrounding the fovea.

**Table 4:** Results of statistical comparison of total retinal thickness (RT) and total retinal volume (RV) values of hemodialysis patients (study group) to healthy control patients. Values are presented as median (25% quartile; 75% quartile). *p* values ≤ 0.05 are highlighted in bold.

Parameter	Study Group	Control Group	*p* Values
Total RT (µm)	295.90 (285.20; 310.00)	312.44 (304.90; 328.80)	**<0.01**
Total RV (mm^3^)	2.67 (2.58; 2.80)	2.85 (2.82; 2.96)	**<0.01**

RT = retinal thickness, RV = retinal volume.

## Data Availability

Not applicable.
